# Low and facultative mycorrhization of ferns in a low-montane tropical rainforest in Ecuador

**DOI:** 10.1371/journal.pone.0326712

**Published:** 2025-07-08

**Authors:** Jennifer Michel, Marcus Lehnert, Martin Nebel, Dietmar Quandt

**Affiliations:** 1 Department of Plant Biodiversity, Bonn Institute of Organismic Biology (BIOB), University of Bonn, Bonn, North Rhine-Westfalia, Germany; 2 Plant Sciences, TERRA Teaching and Research Centre, Gembloux Agro-Bio Tech, University of Liège, Gembloux, Wallonia, Belgium; 3 Geobotanik und Botanischer Garten, Martin-Luther-Universität Halle-Wittenberg, Halle (Saale), Saxony-Anhalt, Germany; 4 Staatliches Museum für Naturkunde Stuttgart, Zentrum für Biodiversitätsforschung, Stuttgart, Baden-Württemberg, Germany; Friedrich Schiller University, GERMANY

## Abstract

Arbuscular mycorrhizal fungi (AMF) are amongst the most studied obligate plant symbionts and regularly found in terrestrial plants. However, global estimates of AMF abundance amongst all land plants are difficult because i) the mycorrhizal status of many non-commercial, wild plant species is still unknown, ii) numerous plant species engage in facultative symbiosis, meaning that they can, but do not always do, associate with mycorrhiza, and iii) mycorrhizal status can vary within families, genera, and species. To gain deeper insights to the distribution of the plant-AMF symbiosis we investigated the mycorrhizal status in some of the oldest lineages of extant vascular plants, Polypodiophytina (ferns) and lycophytes, in one of the hotspots of natural plant diversification, the tropical rainforest. Providing a new data set of AMF abundance for 82 fern species representing 19 families, we hypothesized that (1) AMF would be found in 60–80% of the studied plants and (2) plant species with AMF symbionts would be more abundant than non-mycorrhizal species. Both hypotheses were rejected while the following observations were made: (1) AMF occurred in 30.5% of studied species, representing 63% of the studied fern families, (2) AMF colonisation was not correlated with species abundance, (3) a small proportion of AMF-hosting ferns was epiphytic (6%) and (4) mycorrhization was inconsistent among different populations of the same species (facultative mycorrhization). While these observations align with previous studies on ferns, they emphasise that mycorrhization is not a taxonomic trait and underscore the challenges in estimating the global abundance of AMF. In addition, the occurrence of AMF in epiphytic plants and no net benefits of AMF for plant abundance indicate that the mycorrhization observed in this study likely comprises the commensalism to parasitism range of the symbiosis spectrum.

## Introduction

Arbuscular mycorrhizal fungi (AMF) are amongst the most studied plant symbionts and it is proposed that AMF occur amongst 80% of plant species globally [1,2]. The inter-kingdom interaction between the arbuscule-forming fungi from the phylum *Glomeromycota* and land plants is thought to be long-standing, with records of arbuscular structures inside plant roots dating back to 400-Million-year-old fossils [3,4]. The Devonian period (415–360 Ma) was characterized by an explosion in the diversity of land plants [5] and the survival of the AM fungi-plant-relationship from the Devonian to present day times suggests an evolutionary benefit for both partners. The general principle of the textbook example of a mutualistic symbiosis is that the plant transfers carbon (C) from its photosynthetic activity to the rhizosphere, where C is exchanged with fungi. Fungi in turn help the plant to obtain nutrients like phosphorous (P) or nitrogen (N) from the soil matrix, which the fungi can release from soil minerals through mechanical or enzymatic activity of their hyphae [6–8]. However, symbiotic interactions are nuanced and not always mutually beneficial.

The word “symbiosis” derives from the Greek “symbiōsis”, a combination of “syn”, meaning “with” and “bios”, meaning “life”, together meaning “state of living together” [9]. When both partners benefit from the interaction, the relationship is termed “mutualism”. When one organism lives off another at the other’s expense, it’s called “parasitism”. When one species obtains food or other benefits from its symbiont, without either harming or benefiting the latter, it’s called “commensalism”. The character of a symbiotic interaction is case and context dependent, often a trade-off between dependence and benefit [10–13]. For example, the plant-AMF symbiosis involves mutualism, commensalism, parasitism and everything in-between [14], but in popular literature the term “symbiosis” often refers to only the narrow fraction of the whole symbiosis spectrum that consists of mutualism [15]. Similarly, Karst et al. (2023) highlight an overly positive citation bias in studies investigating common mycorrhizal networks in forests [16] and in their critical revision of both agronomic and mycorrhizal literature Ryan and Graham (2018) show that no consistent link exists between yield benefits and AMF colonisation [17]. AMF studies are also sensitive to geographical biases, with more AMF investigations conducted in the Northern hemisphere on domesticated plants [18] and with unknown mycorrhization status for still 99% of plant species [19]. This likely led to an overestimation of the global occurrence of the AMF-plant-symbiosis and deep insights to the evolutionary trajectory of AMF are limited as the interaction is seldom studied at the hotspots of natural plant diversification which are located in the Global South, particularly along the Andean-Amazonian foothills [19–21]. Moreover, early AMF studies focussed on histological quantification of mycorrhizal structures, but lacked links to functioning or activity of AMF, and often also had no negative controls, which doesn’t allow conclusions about the character of the symbiosis along the mutualism-parasitism continuum [14,22]. For *Plantago* for example, it has been shown that AMF abundance decreases with phylogenetic branch length, with indication that the reduction in AMF colonisation might be driven by energetic costs [23]. And for ferns, AMF-colonisation is often found for a more moderate proportion of 44–54% of species, with many of them engaging in facultative symbiosis [24,25]. Higher colonisation rates have also been reported for fern species (78%), but interestingly while mycorrhizal species were more abundant, non-mycorrhizal species showed higher growth rates and higher leaf nutrient concentrations [26]. A further important consideration for an evolutionary understanding of the AM fungi-plant-relationship is the fact that all AMF are plant-obligate symbionts, but only few plants are mycotroph [27]. Therefore, occurrence and functional and ecological integrity of AMF remain controversial topics.

This study extends the sparse data of AMF abundance in the Southern Hemisphere by providing new empirical data further quantifying the presence of AMF amongst ferns and lycophytes. The study focusses on fern species registered in a previous biodiversity survey in a low-montane tropical rain forest in eastern Ecuador [28]. Ferns are an evolutionary old division and amongst the first vascular land plants, who appeared around 400 million years ago in the Devonian [29]. Later in the Cretaceous, a fern radiation occurred giving rise to most modern fern families [30]. Ferns then became abundant floral elements in both temperate and tropical forests [31,32]. Given their wide distribution and versatility, ferns are also recognised as good indicators of overall biodiversity [33,34]. In addition, ferns thrive in the highly competitive tropical hotspots of natural plant diversification despite the shade underneath the rainforest canopy and the highly weathered nutrient-poor soils, which makes them an ideal study target to investigate plant-AMF symbiosis [35–38].

In this study, two specific hypotheses were tested assuming the fern-AMF symbiosis would exhibit the classical properties assumed for land plants:

(H1): AMF will be highly abundant amongst ferns (60–80%).

(H2): Species with regular AMF symbiosis will be more abundant at plot level.

## Materials & methods

### Study area

**Fig 1:**
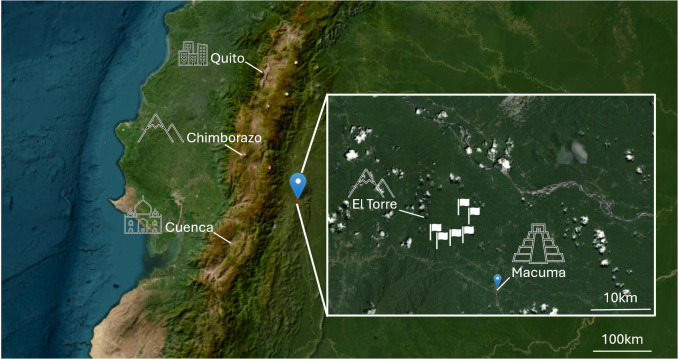
Location of the study area in the lowlands of the Eastern Andes in Ecuador in the province Morona Santiago (via [41]).

The study area is located in eastern Ecuador in a remote and undisturbed part of the western Amazonian rainforest on the northern extensions of the foothills of the Cordilliera de Cutucú (Fig 1). The area is situated east of the national road E45 between El Puyo and Macas, between the rivers Pastaza and Macuma heading towards the township of Macuma (S02º06.664’ W077º44.334’). The region is influenced by the high-pressure area over the central Amazon basin throughout the entire year and receives between 2500 mm and 4000 mm rainfall per year at moderate temperatures around 20°C [28]. All sampling sites are located below 1500 m elevation and the soils are mostly entisols and inceptisol of low pH with considerable variation in exchangeable cations (Table 1). The climatic conditions remain relatively stable during the year and provide plants with an almost continuous vegetation period, making it a recognised hotspot of biodiversity and a habitat very suitable for ferns [39,40].

**Table 1:** Geochemical characterisation for the six studied plots.

PLOT	Latitude	Longitude	Elevation (m)	Soil type	pH_H2O_	C (%)	N (%)	C:N	Al	Ca	K	Mg	Mn	Na	∑ Exchangeable cations
									μmol g^-1^ dry weight soil						
WIS 1	S 02º 06.803’	W 077º44.582’	665	Enti/Inceptisol	5.26	5.50	0.50	10.97	4.98	186.52	2.40	11.54	0.33	0.66	206.43
WIS 2	S 02º 06.845’	W 077º44.587’	658	Alfisol	6.82	7.95	0.69	11.48	0.00	391.35	3.36	19.29	0.05	0.58	414.64
WIS 3	S 02º 06.364’	W 077º45.788’	996.5	Enti/Inceptisol	4.36	5.13	0.44	11.57	47.80	11.11	0.67	1.04	0.44	0.29	61.35
WIS 4	S 02º 06.361’	W 077º45.788’	1002.5	Enti/Inceptisol	4.02	7.32	0.55	13.10	60.66	1.17	0.53	0.59	0.07	0.26	63.29
WIS 5	S 02º 06.000’	W 077º44.543’	679.5	Enti/Inceptisol	4.18	7.39	0.57	12.89	69.86	32.81	2.43	10.37	0.47	0.28	116.22
WIS 6	S 02º 05.882’	W 077º44.529’	688	Enti/Inceptisol	4.26	7.99	0.66	12.04	80.10	30.31	1.30	14.97	0.71	0.10	127.49

Abbreviations: C = Carbon, N = Nitrogen, C:N = carbon to nitrogen ratio, Al = aluminium, Ca = calcium, Fe = iron, K = potassium, Mg = magnesium, Mn = manganese; Na = sodium.

### Inclusivity in global research

The study took place on the territory of the Shuar community of Wisuí who kindly granted us access to their land and safely guided us through the jungle. Research permits (No. 03–2012-Investigación-B-DPMS/MAE) were issued by the Ministerio del Ambiente, Dirección Provincial del Morona-Santiago, Ecuador. Additional information regarding the ethical, cultural, and scientific considerations specific to inclusivity in global research is included in the supporting information S1 Text.

### Biodiversity survey and root collection

Ferns and their roots were collected in six plots established on the lower slopes of the mountain ridges (658 m – 1002 m). Each plot measured 20 m × 20 m (400 m^2^), and an additional collection was made on the mountain top of El Torre (1370 m). All fern species occurring within a plot were documented photographically and their life form was recorded (terrestrial, hemi-epiphytic, epiphytic). Plant species were assigned following morphological identification and taxonomy was cross-checked at the herbarium in Quito (QCA) and against PPGI (2016) and Hassler et al. (2022) [42,43]. Where available, four replicate plants were collected of each species in each plot, and plant material was conserved for distribution to the different collaborating herbaria (QCA, MAC, STU, BONN). For each plot, alpha diversity was determined (number of different species per plot), and for each species their abundance (number of individuals of a species per plot) and relative abundance (the number of individuals of a species per plot relative to the total number of individuals per plot) were determined. Root samples were collected from each specimen. All root samples were first carefully cleaned and subsequently stored in 70% ethanol in Eppendorf tubes. In total, n = 97 root samples were collected for AMF quantification (supporting information S2 Table). Terrestrial and epiphytic ferns had similar sampling size (n = 44 terrestrial, n = 11 hemi-epiphytic, n = 42 epiphytic). In addition to the biodiversity survey and the root samples, a composite sample of approximately 150 g of the upper soil horizon was taken in each plot for soil biochemical analysis.

### Root sample preparation and mycorrhiza documentation

To investigate presence/absence of AMF structures in root samples, the protocol after Grace & Stribley (1991) was used [44]. Root samples were first transferred from the ethanol into 10% KOH and left for clearing 24h at 60°C. Then, root material was rinsed with distilled water twice and acidified with 10% lactic acid. As a dye, Aniline Blue WS (Sigma Aldrich, Missouri, US) was prepared in 0.05% lactic acid by dissolving 0.125 g solid Aniline blue in 125 ml distilled water and 125 ml lactic acid (0.125g/250 ml). Roots were analysed in a two-step process: First, aliquots of 10 cm total root length of each sample were exposed to the staining solution for 45 min at 60°C. After washing with distilled water, these samples were used for screening and the detection of fungal hyphae. For this purpose, roots were cut into 10 pieces of 1 cm length each. The 1 cm fragments were then aligned on microscope slides and gently prepared as squeeze fractions with the coverslip. In a second step, another aliquot of each root sample was analysed in cross sections to verify the localisation of arbuscules within parenchymal cells. For this purpose, untreated root material of each specimen was taken out of the ethanol and sliced into at least ten thin crosscuts, which were placed upon microscope slides. The thin cuts were covered with 10% KOH for 60 min and after acidification with 90% lactic acid, the procedure finished with the application of the Aniline Blue dye. All preparation steps were incubated on a heating plate at 45°C and constantly covered with a cover slide. Liquids were exchanged by pipetting them to one side of the coverslip while holding paper to the other side using capillary action. Visual inspection of both lateral squeeze sections and crosscuts was performed with a binocular stereomicroscope (Leitz F193, Leica Microsystems, Wetzlar, Germany; magnification: 16x), a light microscope (Leitz Laborlux S, Leica Microsystems, Wetzlar, Germany; magnification: 40x – 400x) with an Olympus D700 camera (Olympus Corporation, Tokyo, Japan) and a confocal microscope and its corresponding camera and software (VHX– 500I, Keyence, Osaka, Japan; magnification: 250x – 2500x). Each sample was than classified as either “mycorrhizal” or “non-mycorrhizal”. Species that occurred in several plots were classified as “facultative mycorrhizal” if they were “mycorrhizal” in one plot, but “non-mycorrhizal” in another.

### Data analysis

To test whether AMF root colonisation differed between plant growth forms (terrestrial, hemi-epiphytic or epiphytic), and if relative abundance of fern species was influenced by mycorrhization (mycorrhizal, non-mycorrhizal, facultative mycorrhizal) we used analysis of variance. To test whether fern α-diversity was determined by the interaction between soil factors (cation exchange capacity or pH) and AMF root colonisation we used analysis of co-variance with α-diversity as dependent variable and respective soil parameter and AMF colonisation as covariates. All statistical analysis was carried out using R 4.4.1 with the additional packages *car*, *ggplot2* and *multcomp* [45–48].

## Results

### AMF at family and species level

**Fig 2:**
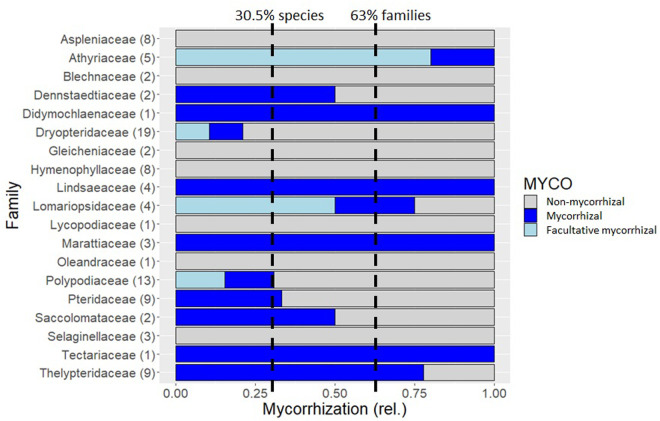
Abundance of AMF amongst 19 fern families of the Ecuadorian low-montane tropical rain forest. Number in brackets behind family name indicates number of samples investigated within each family, total number of samples n = 97, corresponding to n = 82 species. Dotted lines indicate average root colonisation with AMF across all investigated fern species (30.5%) and across all investigated fern families (63%) respectively.

Arbuscular mycorrhiza fungi were found colonizing individuals of twelve out of nineteen fern families (Fig 2). Except for Marattiaceae (Marattiopsida), all families are encompassed by the class Polypodiales [42]. Reflecting the overall biodiversity in the study area, there was variability in the number of samples per family. For example, only one sample corresponds to the family Didymochlaenaceae while 19 samples represent Dryopteridaceae. The highest rate of mycorrhization was found for Lindsaeaceae, Marattiaceae and Tectariaceae where all investigated samples presented AMF. Amongst those families well represented, strong mycorrhization was also found for Thelypteridaceae where six out of eight species associated with AMF. On the other hand, AMF were completely absent for all samples of Aspleniaceae, Blechnaceae, Gleicheniaceae, Hymenophyllaceae, Lycopodiaceae, Oleandraceae and Selaginellaceae. Amongst those families well represented, low mycorrhization was also observed for Dryopteridaceae where two out of 15 species were found with AMF (*Elaphoglossum ensiforme* (epiphytic) and *Polybotrya polybotryoides* (hemi-epiphytic)), and for Polypodiaceae, where two out of nine studied species associated with AMF (*Campyloneurum phyllitidis* and *Serpocaulon adnatum* (both hemi-epiphytes)). A total of 82 species was investigated, with 25 consistently found associated with AMF (30.5%). Amongst those species present in more than one plot, five species were found with facultative mycorrhization, namely *Diplazium ambiguum* (Athyriaceae), *Diplazium expansum* (Athyriaceae)*, Microgramma thurnii* (Polypodiaceae)*, Nephrolepis rivularis* (Lomariopsidaceae) and *Polybotrya fractiserialis* (Dryopteridaceae).

### AMF occur in plants of all habitats but do not increase plant species abundance

**Fig 3:**
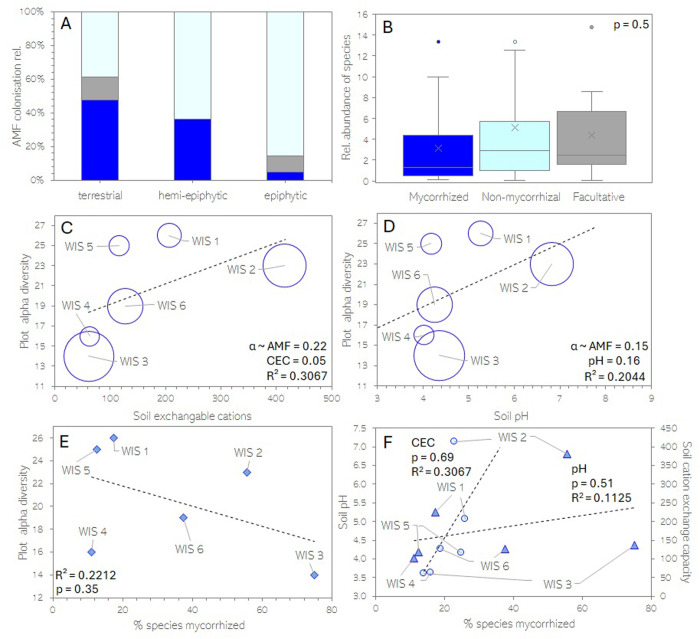
Relationships between (A) AMF colonisation and plant habitat (terrestrial, hemi-epiphytic or epiphytic), (B) Relative abundance of fern species and their AMF colonisation (mycorrhizal, non-mycorrhizal, sometimes mycorrhizal (facultative)), (C) Fern α-diversity at plot level, soil exchangeable cations (CEC) and degree of AMF-colonisation (indicated by the size of the circle), (D) Fern α-diversity at plot level, soil pH and degree of AMF-colonisation (indicated by the size of the circle), (E) Fern α-diversity at plot level and degree of AMF-colonisation and (F) Soil pH (triangles), cation exchange capacity (circles) and degree of AMF-colonisation.

Mycorrhizal colonisation was quantified for n = 97 root samples where each sample was assigned to a habitat type as either terrestrial, epiphytic or hemi-epiphytic. Overall, 33% of all samples evidenced mycorrhizae. Terrestrial ferns species had significantly higher colonisation levels (53.5%) than epiphytic ferns (7.3%). The plants of hemi-epiphytic habitats presented an intermediated level of mycorrhization of 36.4% (Fig 3A). There was no correlation between the mycorrhizal status of a fern species (mycorrhizal, non-mycorrhizal or facultative mycorrhizal) and the species’ relative abundance in a plot (Fig 3B). In an analysis of co-variance, fern alpha diversity remained unrelated to AMF abundance, but soil cation exchange capacity was correlated with fern species richness (p = 0.047), while soil pH was not (p = 0.16) (Fig 3C, D). There was also no linear correlation between species richness and the degree of mycorrhization in that plot (Fig 3E) or between mycorrhization and soil parameters (Fig 3F).

## Discussion

### How abundant are AMF amongst land plants and amongst ferns?

Most AMF research addresses economically important crops and culturally valuable species, while significantly less is known about AMF in other plant groups [1,2,18]. Albornoz et al. (2021) proposed that abundance of AMF is possibly globally overstated as the mycorrhizal status of 99% of plant species is still unknown [19]. AMF-abundance can also easily be overestimated because “mycorrhization” is not a taxonomic trait, meaning that AMF colonisation can vary both amongst different species within one genus or family, as well as amongst individuals of the same plant species in the same location [19,49,50]. In addition to the de facto variability of AMF occurrence across time and space caused by environmental and seasonal drivers, there may also be differences in AMF-estimates amongst different studies due to sampling and observation biases which affect both histological and molecular quantification methods [22,51]. Understanding mycorrhization can be particularly difficult in rare species when replication is impeded by their low abundance, as was the case for several families in this study where the floral composition of the plots was characterised by high uniqueness. While generalisations are difficult to draw from such data sets alone, they provide important information to fill the gaps in global biodiversity research [20,52]. Overall, it is assumed that AMF associate frequently with modern world angiosperms, with several studies suggesting that 80% of plant species globally engage in AMF-symbiosis [1,2]. In line with the observations in this study, AMF association however seems to be less common in older linages of vascular plants such as ferns [53]. Here, we found 30.5% of fern species associated with AMF, representing 63% of the studied families. These observations are in line with a previous study that investigated AMF symbiosis in ferns in Southern Ecuador and found AMF in 29.1% of terrestrial species, equivalent to 48.49% of terrestrial samples [54] but lower compared to a global analysis reporting mycorrhization in 62% of fern species [53]. It has been suggested that the abundance and reliance on AMF in ferns may have decreased during the evolutionary differentiation of fern genera as they occupied niches where they were more competitive without the fungal symbionts [27,55]. Ferns might therefore rely more on other adaptive traits to compete, such as efficient spore dispersal, rapid growth in high-light conditions, and tolerance to less fertile soils. In addition, reduced dependence on fungal partners could have made ferns more resilient to environmental fluctuations or disruptions that might otherwise affect AMF communities and their dependant hosts.

### Facultative mycorrhization?

Facultative mycorrhization refers to plants that can form mycorrhizal associations with AMF, but do not rely on symbiotic partners for growth or survival. In this study five fern species were found to be facultatively mycorrhizal, namely *Diplazium ambiguum, Diplazium hians, Microgramma thurnii, Nephrolepis rivularis* and *Polybotrya fractiserialis*. Facultative mycorrhization is common among plants and has also been described for ferns [24,49,56]. For example, it has been observed that AMF colonisation in *Struthiopteris spicant* (Blechnaceae) increases with higher light intensity, likely related to an increased availability of photosynthetically fixed carbon compounds which are a main trademark in mutualistic symbiosis [1,56]. In addition, AMF have low taxonomic diversity, and the community composition of AMF is dynamic as a function of light conditions, soil nutrient levels and seasonal weather patterns, which can influence AMF-host affinity [50,56–58]. Mycorrhization thus seems to be more driven by environmental factors than by host identity, which aligns with our finding that plant α-diversity was independent of AMF colonisation (Fig 3C–E). Accordingly, it has been observed in both pot and field experiments that AMF association in ferns can be independent of soil nutrient status [26,59] but instead seems to correlate with soil pH [50,60]. In this study soil cation exchange capacity explained fern species richness better then AMF abundance or pH, but there was no correlation between AMF and soil pH (Fig 3F). Given the variable relation between soil factors and AMF abundance and that the occurrence of AMF is often unrelated to the host plant species’ abundance suggests a more commensalistic character of the symbiosis. Variability amongst studies possibly also links to the occurrence of facultative mycorrhization. To further constrain the relationship between plant species abundance and mycorrhizal status larger sample sizes and consideration of other covarying factors such as plant symbionts other than AMF are needed. Future studies could also address how common facultative mycorrhization is in angiosperms, as this type of symbiosis adds important nuance to global upscaling estimates of mycorrhization amongst plant species and families [2,49]. The environmental conditions and season during which abundance studies are conducted can also influence recorded mycorrhizal colonisation rates [50]. As temporally-limited observations of AMF-associations may not reflect the long-term ecological significance of AMF in ecosystems, further studies investigating the functioning of AMF in natural ecosystems are crucial.

### What do AMF do in the roots of epiphytic plants?

This and previous studies recorded AMF associations in epiphytic plants [25,26,53,54]. AMF associations in epiphytic plants are intriguing, as these plants live on other plants high up in the canopy rather than in the soil. In some cases, canopy habitats can accumulate organic matter, creating a form of “canopy soil” [61]. In these mini-ecosystems, AMF could facilitate nutrient transfer from decomposing materials, functioning similarly to the mutualistic symbiosis described for terrestrial ecosystems, but within the canopy structure. It is also possible that the AMF communities found in epiphytic ferns are in a dormant state [62]. Dormancy is common in AMF as it allows them to survive periods when environmental conditions like moisture and nutrient scarcity impede active growth or symbiosis [1]. Finally, it might also well be possible that AMF in epiphytic plants are at the parasitism end of the symbiosis spectrum simply seeking for survival [11,14]. The presence of AMF in epiphyte roots also raises the question how the fungi arrived so high up in the canopy. AMF spores could reach epiphytic plants through wind dispersal, rain splash, or animal movement. Tree trunks and branches can act as conduits for spores to reach higher levels in the canopy, where they might form symbioses with epiphytes. Once in the canopy, AMF could sustain themselves on organic materials and humus, allowing them to persist and associate with epiphytic plants [63,64]. They may also be artefacts from plants which grow in the canopy at the time of sampling, but retain or once had a connection to the soil [25,65]. Future studies could try to answer the question what vertical distribution patterns AMF exhibit along trees and their epiphytes, ideally making use of molecular techniques.

### Are all AMF always actively involved in mutually beneficial symbiosis?

The observations of facultative mycorrhization and mycorrhization of epiphytic plants highlight the broad character of symbiosis in natural ecosystems, which spans a large spectrum from mutualism, over commensalism to parasitism, and certainly AMF associations with plants are not always mutually beneficial [14,15]. Without further measurements, the sheer presence of AMF inside plant roots does not allow conclusions about whether the co-occurrence of these fungi inside plant structures has benefits for either of the two organisms. Hence, it is possible that AMF have been hitch-hiking plants since the Devonian, probably mostly as commensalists. To better understand the symbiosis, observational data of root colonisation needs to be paired with plant nutrient uptake measurements and/or plant species abundance, with the relevant controls, to infer details about the character of the symbiosis. In their critical revision of both agronomic and mycorrhizal literature, Ryan and Graham (2018) show for example that no consistent link exists between yield benefits and AMF colonisation, and AMF are not required for optimal plant phosphorous nutrition [17]. Sometimes AMF keep nutrients away from the plant, and sometimes reverse flow keeps water away from the plant [66,67]. In field studies, especially commercial AMF inoculum containing only a reduced number of fungal species seldom work [68]. Competition between AMF species is also commonly reported [69] and it has been shown that under elevated CO_2_ AMF can cause substantial losses of soil carbon [7]. AMF can also negatively impact their fellow soil organisms by keeping carbon away from them [70]. Another benefit attributed to AMF is their contribution to the formation and stability of soil aggregates [71], but this function can also be carried out by a plethora of different soil organisms [72], plant root mucilage [73], or by purely geochemical interactions between molecules in the soil matrix [74]. In line with these critical aspects, our results also call for a more nuanced interpretation of AMF-symbiosis and invite the possibility to also consider a not-mutualistic role of AMF, which could be further clarified by more studies investigating AMF abundance in undisturbed natural environments, and by making clear links between presence and functionality of AMF, for example using stable isotope probing [75,76].

## Supporting information

S1 Text Inclusivity in global research statement. (DOCX)

S2 Table Table of empirical observations. (XLSX)
